# Functional metabolomics as a tool to analyze Mediator function and structure in plants

**DOI:** 10.1371/journal.pone.0179640

**Published:** 2017-06-22

**Authors:** Celine Davoine, Ilka N. Abreu, Khalil Khajeh, Jeanette Blomberg, Brendan N. Kidd, Kemal Kazan, Peer M. Schenk, Lorenz Gerber, Ove Nilsson, Thomas Moritz, Stefan Björklund

**Affiliations:** 1 Department of Medical Biochemistry and Biophysics, Umeå University, Umeå, Sweden; 2 Umeå Plant Science Centre, Department of Forest Genetics and Plant Physiology, Swedish University of Agricultural Sciences, Umeå, Sweden; 3 Plant-Microbe Interactions Laboratory, School of Agriculture and Food Sciences, The University of Queensland, St Lucia, QLD, Australia; 4 CSIRO Agriculture and Food, Queensland Bioscience Precinct, 306 Carmody Road, St Lucia, QLD, Australia; 5 Queensland Alliance for Agriculture & Food Innovation (QAAFI), University of Queensland, St Lucia, QLD, Australia; Università degli Studi di Milano, ITALY

## Abstract

Mediator is a multiprotein transcriptional co-regulator complex composed of four modules; Head, Middle, Tail, and Kinase. It conveys signals from promoter-bound transcriptional regulators to RNA polymerase II and thus plays an essential role in eukaryotic gene regulation. We describe subunit localization and activities of Mediator in *Arabidopsis* through metabolome and transcriptome analyses from a set of Mediator mutants. Functional metabolomic analysis based on the metabolite profiles of Mediator mutants using multivariate statistical analysis and heat-map visualization shows that different subunit mutants display distinct metabolite profiles, which cluster according to the reported localization of the corresponding subunits in yeast. Based on these results, we suggest localization of previously unassigned plant Mediator subunits to specific modules. We also describe novel roles for individual subunits in development, and demonstrate changes in gene expression patterns and specific metabolite levels in *med18* and *med25*, which can explain their phenotypes. We find that *med18* displays levels of phytoalexins normally found in wild type plants only after exposure to pathogens. Our results indicate that different Mediator subunits are involved in specific signaling pathways that control developmental processes and tolerance to pathogen infections.

## Introduction

Mediator was originally identified in *S*. *cerevisiae* as a novel factor required for transcriptional regulation. Biochemical purification showed that it is a multiprotein complex which integrates signals to regulate expression of genes transcribed by RNA polymerase II (Pol II) [1, 2]. This regulation is achieved through interactions between Mediator and transcriptional regulators (activators and repressors) that bind to specific promoter sequences [3]. Mediator subsequently transfers signals from the transcriptional regulators to the basal transcription machinery by also interacting with Pol II.

Yeast Mediator comprises 25 subunits that can be divided into four modules: Head, Middle, Tail, and a separable, repressive CDK8-kinase module, which reversibly associates with Mediator for regulation of Mediator–Pol II interaction at transcription (re)initiation [4–7] Head interacts with Pol II and stimulates basal transcription. Middle also interacts with Pol II and is involved in processing of signals from Tail to Head while Tail makes direct contact with transcriptional regulators. The CDK8-module is more loosely associated to the other three modules and is involved in transcriptional repression by preventing interaction between Mediator and Pol II. Mediator is evolutionarily conserved and a modular structure has also been suggested for Mediator in mammalian cells [6, 8].

We reported the first biochemical identification of Mediator in plants and showed that *Arabidopsis thaliana* comprises homologs of most yeast and human Mediator subunits [9]. A unique feature for Arabidopsis is that it encodes paralogs for several Mediator subunits such as MED7, MED10, MED15, MED19, MED20, MED22, MED26, MED33, MED35, MED36, MED37, and CycC. Interestingly, we detected unique peptides from several of the paralogs in Mediator purified from Arabidopsis, thus indicating that paralogs are both expressed and built into Mediator [9, 10]. The functions of paralogs are unknown and it is possible that they perform redundant functions. However, the fact that they have been conserved suggests that they might display unique expression patterns in different tissues or during different developmental stages. Such unique expression patterns have for example been described for the Arabidopsis small ubiquitin-like modifier paralogs [11].

While plants lack cells with specialized, defense-related functions, they are able to recognize pathogens for initiation of defense responses. These include synthesis of hormones such as jasmonates, salicylic acid (SA), and ethylene (ET), various pathogenesis-related proteins, and secondary metabolites such as flavonoids and glucosinolates. Jasmonic Acid (JA) is an oxylipin, which acts as a hormone by regulating expression of defense and development related genes. It is produced by oxygenation of linoleic acid present in lipids of membranes, in particular in chloroplasts [12]. Arabidopsides is a unique class of Arabidopsis oxylipins and their levels increase up to 1000-fold upon wounding [13]. SA is a phenolic phytohormone with important functions in plant development and defense, and it is known to act antagonistically with JA [14]. SA plays a role in pathogen resistance by regulating pathogenesis-related proteins and it is involved in Systemic Acquired Resistance (SAR) [15].

Plants use chemical signals and activation of transcription factors (TFs) to coordinate defense signaling networks and to regulate growth, development, and stress responses. Mediator is a likely receiver of such signals since it orchestrates developmental cues and cellular responses to external stimuli such as light, temperature, drought, high salt concentrations, or pathogens [16, 17]. We recently identified three Arabidopsis TFs; DREB2A, ZFHD1, and PHL1 (previously named MYB-LIKE), that interact with the ACtivator Interaction Domain (ACID) of MED25 [17, 18]. They have previously been implicated in stress responses, but we could assign new functions for DREB2A. In addition to its involvement in response to drought, we found that DREB2A is also involved in a light-quality pathway downstream of phytochrome B (PHYB) by interacting with MED25. We hypothesize that also the head subunit MED8 is involved in this pathway since *med8* displays several phenotypes similar to *med25* [16, 17]. Both the *med8* and *med25* mutants are resistant to *Fusarium oxysporum* infections and show a delay of flowering time [16, 19], and we recently reported that also the Mediator head module subunits MED18 and MED20 confer susceptibility to *F*. *oxysporum* in Arabidopsis [20]. Furthermore, it was recently reported that MED14, MED15, MED16, MED19a, and MED21 are involved in plant immunity [21, 22]. Factors acting downstream of Mediator in these processes are in most cases still unknown. However, it was shown that TFs belonging to the MYC and ERF families, which are known to induce JA-associated gene expression, interact with MED25 [23, 24]. This provides a molecular mechanism for down-regulation of JA-associated genes in *med25*.

Here we report on changes in gene expression and levels of metabolites in a set of Arabidopsis Mediator mutants and *dreb2a*. By combining functional metabolic profiling and heat map visualization we show that all mutants display distinct metabolite profiles that differ from wild type (WT; Col-0) and that the metabolite patterns for mutants from each Mediator module cluster together. Functional metabolomic analysis thus provides a new and complementary method to predict the location of unassigned subunits to specific Mediator modules. We show that phenotypes of certain mutants can be explained by changes in gene expression and by the levels of specific metabolites and that MED18 is involved in regulation of genes controlling responses to pathogen infections. Thus, the uninfected *med18* displays levels of metabolites similar to the levels normally found in wild type cells after they have been exposed to pathogens. Our results therefore provide new insights into the function of Mediator in regulating plant metabolism.

## Materials and methods

### Description of mutant lines

The mutants used correspond to the T-DNA insertion lines SALK_102813 (at5g20170, *med17*), SALK_027178 (at2g22370, *med18*), SALK_034955c (at5g12230, *med19a*), SALK_063109C (at1g16430, *med22a*), SALK_001024C (at1g07950, *med22b*), SALK_128011 (at1g23230, *med23*), SALK_129555 (at1g25540, *med25*), SALK_012449 (at3g09180, *med27*), SALK_037570 (at3g52860, *med28*), SALK_023845C (at1g11760, *med32*), SALK_089976 (at3g23590, *med33a*), SALK_063418 (at1g31360, *med34*), and SAIL 365_F10 (at5g05410, *dreb2a*).

### Plant materials and growth conditions

All mutant lines were of the Colombia accession (Col-0) and obtained from the Nottingham Arabidopsis Stock Center (NASC). The Col-0 is named WT throughout the text. T-DNA insertions were checked by PCR genotyping using T-DNA left border and gene-specific primers designed by the Salk Institute Genomic Analysis Laboratory (SIGnAL) (http://signal.salk.edu/tdnaprimers.2.html) using default conditions. Homozygote lines for each mutant were then identified, except for Med17, which could only be identified as a heterozygote line (S1 Table). The expression levels of each subunit mRNA in the corresponding mutant line relative to the levels in WT was determined by RT-PCT (S1 Table) using specific primers (S2 Table) 15 plants per genotype were grown in long day (LD; 16h light/8h dark) conditions using a growth chamber. Data collection and measurements were performed as described [25].

### Data collection, measurements and different treatments

Plants were grown in a mix (2:1) of soil and vermiculite in individual pots. 15 plants per genotype were cultivated in a growth chamber with 60–70% relative humidity and a day/night cycle (16hr-23°C/8h-20°C) at around 110 μmol.m^-2^.s^-1^. The total number of leaves (rosette leaves plus cauline leaves) was counted after the main stem had bolted 10 cm. Data are given as averages ± SD for 3–4 individual experiments. For the microarray and metabolomic experiments, leaves of *med18*, *med25*, and WT were collected at the bolting time of WT. The experiments were repeated at least 3 times.

### RNA extraction

Leaves of plants (mutants and WT) grown in LD conditions were collected, frozen and stored at –80°C until extraction. Total RNA was isolated using the RNeasy Plant Mini Kit (Qiagen Nordic, Sollentuna, Sweden). For each sample the DNase treatment was performed during the extraction according to the Qiagen protocol with RNase-free DNase I (Qiagen) to eliminate genomic DNA contamination.

### ATH1 Affymetrix microarrays analysis

GeneChip microarrays were performed using 10 μg of total RNA per sample as described in the Affymetrix GeneChip technical analysis manual (Affymetrix UK Ltd, High Wycomb, UK). RNA integrity was checked by an Agilent Bioanalyzer. Microarray experiments were performed in the Affymetrix core lab of Nottingham Arabidopsis Stock Centre, Loughborough, UK. Normalization of raw intensities across all probe sets was performed in R language using Robust Multi-array Average (RMA) algorithms and using Bioconductor software (http://www.bioconductor.org). Three replicates of independently grown material were used. The microarray data presented in this publication have been deposited to the ArrayExpress EMBL-EBI (Accession: E-MTAB-5748).

### Metabolite extraction

Fresh leaves of the different mutants were harvested at the bolting time of WT and immediately frozen in liquid nitrogen. 20 mg of fresh weight leaves were extracted according to Gullberg et al [26]. 1 mL of the extraction mixture (chloroform:methanol:water; 20:60:20, v/v/v) was added to each sample in 1.5 mL tubes on ice. After adding a 3 mm tungsten carbide bead (Retsch GmbH & Co. KG, Haan, Germany) to each tube they were shaken at 30 Hz for 3 min in a MM 301 Vibration Mill (Retsch GmbH & Co. KG, Haan, Germany). The beads were removed before centrifugation for 10 min at 14,000 rpm in a Mikro 220R instrument (Hettich, Zentrifugen). The supernatant from each tube (200 μL) was transferred to a 250 μL micro vial (Chromatol Ltd) and evaporated to dryness in a miVac quattro concentrator (Barnstead genevac) without heating. One unique extraction replicate was performed for each sample.

### UHPLC-TOFMS analysis

200 μl of each sample from the metabolite extractions were dried, dissolved in 20 μl methanol and then diluted with 20 μl water containing 0.1% formic acid. The metabolomics analyses were performed using UHPLC-TOFMS (from Waters, Milford, MA USA). The chromatographic conditions and MS analysis in positive mode were performed as described previously [27]. The LC-Ms analysis was performed with a randomized sample run-order. A two-step strategy was used for data processing of the MS files. Prior data processing the MS-files were converted to NetCdf format, and thereafter the NetCDF-files were converted to XCalibur-format by XCalibur file converter (Thermo Fisher Scientific, Bremen, Germany). The first step of the data processing was peak picking and alignment which were done by the software Sieve 1.3 (Thermo Fisher Scientific). The peaks, i.e. putative metabolites, considered to be [M+H]^+^ ions were manually inspected and reintegrated using an in-house script in MATLAB, version 8.4.0, R2014b (The Mathworks, Inc.). The integrated peak area for each putative metabolite was used for quantification. The Metabolomics data have been deposited to the EMBL-EBI MetaboLights database (identifier MTBLS450). The complete dataset can be accessed at http://www.ebi.ac.uk/metabolights/MTBLS450

### Metabolite structural identification

Putative metabolites detected by UHPLC-TOFMS and from statistical analyses considered of interest were identified using a LTQ/Orbitrap XL mass spectrometer (Thermo Fisher Scientific, Bremen, Germany). The extracts were separated by a Thermo Accela LC system, using an Acquity column (2.1×100 mm, 1.7 μm C18 at 40°C) and the same gradient as for the UHPLC-TOFMS analysis. The MS analyses were performed by tandem mass spectrometry. Centroid mass spectra of positive ions were collected in the Orbitrap mass analyzer, with a target mass resolution of 30,000 at m/z 400 and a data dependent MS^2^ scan using the higher energy collision-induced dissociation cell (HCD) with target mass resolution of 15,000 at m/z 400. External mass calibration was performed according to the manufacturer’s guidelines. Most of the glucosinolates were analyzed in negative mode in a parallel analysis.

### Statistical analysis

PCA and SIMCA were performed using the software SIMCA version 13.0 (Umetrics AB, Umeå, Sweden). For OPLS-DA, in-house code was created in MATLAB, version 7.11.0, R2010b (The Mathworks, Inc.). All data were column centered and scaled to unit variance prior to OPLS-DA modelling. Class balanced OPLS-DA models together with boot-strapping were used to discriminate wild-type from mutants. Hierarchical Cluster Analysis (HCA) using Ward’s method and heat-map visualization were performed in the statistical programming language R (R Development Core Team 2009). For details about data analysis, see Pinto et al. [28].

### Expression of recombinant proteins

The pETDuet-1 plasmid (Novagen), which contains two multiple cloning sites (MCS), was used for co-expression of proteins. The pETDuet-*MED25*-S plasmid was constructed by ligation of the *MED25* cDNA into the BglII/KpnI site of MCS2. An S-tag was included at the C-terminus of MED25. A MED20-MED18 co-expression plasmid was made by introducing a ribosome binding site upstream of MED18 and using sequence and ligation-independent cloning (SLIC). MED20-MED18 was cloned into BamHI/NotI of pETDuet-*MED25*-S with a 6xHis tag added to the N-terminus of MED20). The plasmids (pETDuet-*MED25*-S and pETDuet-*MED25*-S/6 x his *MED20*-*MED18* were transformed into *E*. *coli* strain BL21-CodonPlus (DE3)–RIL competent cells (Stratagene). Bacteria were grown in auto induction medium at 37°C for 3 hours and then shifted to 30°C overnight.

### Pull-down experiments

5 ml overnight cultures of Med25-S and Med25S/6 x His Med20-Med18 were harvested by centrifugation and re-suspended in 2.5 ml of lysis/wash buffer (50 mM NaH_2_PO_4_, 150 mM NaCl, 10 mM beta-mercaptoethanol, 30 mM imidazole; pH 8) supplemented with protease inhibitors. Cells were lysed by sonication and the lysates were cleared by centrifugation at 20,000 rpm. 50 μl of Ni-NTA beads were added to 1 ml of each lysate and incubated at 4°C for 20 minutes. The beads were collected by centrifugation and washed three times with 1 ml of lysis/wash buffer. Proteins bound to the beads were eluted in 100 μl elution buffer (50 mM NaH_2_PO_4_, 150 mM NaCl, 10 mM beta-mercaptoethanol, 400 mM imidazole). 5 μl from each lysate and 10 μl from each eluate were separated on a 10% SDS-PAGE and immunoblotted with S-protein HRP conjugated antibodies (Novagen).

## Results

### Metabolic profiling of Mediator mutants as a method to assign Arabidopsis subunits to specific modules

**Fig 1:**
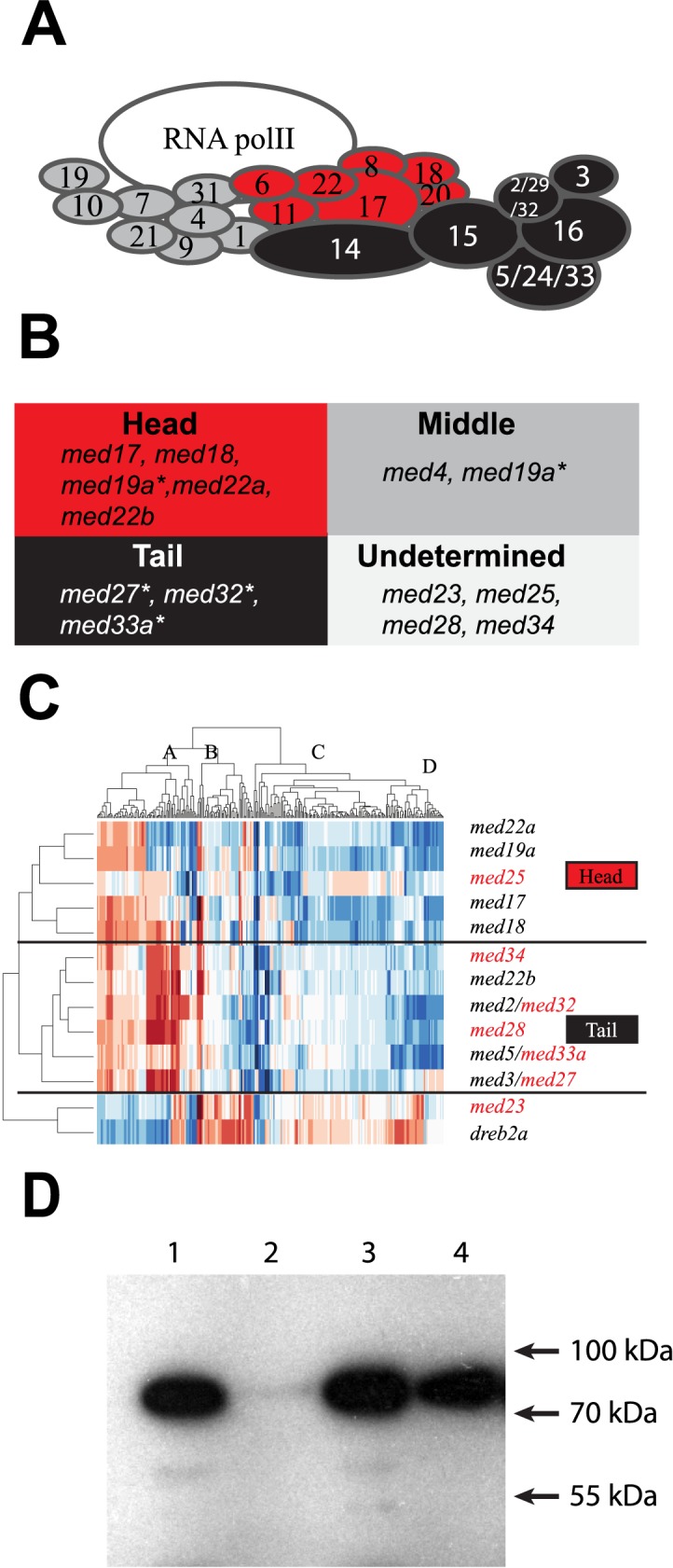
Metabolomic and biochemical analyses of the Arabidopsis Mediator complex. (A) Schematic representation of Mediator in Arabidopsis based on a *S*. *cerevisiae* model and sequence homologies. Subunits from different modules are colored in red (head), dark grey (middle) and black (tail). a and b represent subunits encoded by paralog genes. (B) Mediator mutants included in this study. Unassigned Arabidopsis-specific subunits are labelled in light grey. Asterisks mark mutants in subunits that have only been assigned to a specific module based on sequence homologies with yeast Mediator subunits (MED2/MED32, MED3/MED27/, and MED5/MED33a) or have been placed in different modules in different reports (MED19). (C) Global analysis and visualization of the LC-TOF MS (+) dataset. The OPLS-DA analysis was performed between each mutant line and WT. The hierarchical cluster analysis was performed using the predictive loading vector from the OPLS-DA. The relative abundance of each metabolite per sample is described in S3 Table. A–D represents the metabolite groupings (listed in S4 Table). The metabolite annotation is listed in S5 Table. Red lines represent metabolites that are increased and blue lines represent metabolites that are reduced relative to WT. Mutants labelled in red represent subunits that are specific to Arabidopsis and higher eukaryotes, and have not been assigned to a specific module previously. (D) MED25 interacts with the MED18/MED20 head module dimer. S-tagged MED25 was expressed alone (lanes 1, 2) or together with 6 x his-tagged MED18 and 6 x his-tagged MED20 (lanes 3, 4). Protein extracts from each strain was bound to Ni-NTA beads, washed and eluted as described in Materials and Methods. Proteins from the loads (lanes 1, 3) and eluates (2, 4) were immunoblotted with S-protein HRP conjugate antibodies.

To reveal global functions of Mediator in Arabidopsis, we used LC/TOF-MS metabolite profiling of 12 Mediator subunit mutants (*med17*, *med18*, *med19a*, *med22a*, *med22b*, *med23*, *med25*, *med27*, *med28*, *med32*, *med33a*, and *med34*; Fig 1A and 1B). We also included *dreb2a* since it shows an early flowering phenotype opposed to *med25*, and because the DREB2A protein interacts with MED25 [17, 18]. Sampling of each mutant was performed at bolting time (stage 5.10) to avoid effects due to differences in the timing of entry into specific developmental stages between mutants (S1 Fig). As previously reported, we confirmed that both *med18* and *med25* were delayed in flowering and we found that this phenotype is caused by an extended vegetative phase in both mutants. All 13 mutants displayed significant changes in specific classes of metabolites compared to WT (Fig 1C, S2 Fig; S3–S6 Tables). We subjected the predictive loading vectors p_p_ from bootstrapped Orthogonal Projections to Latent Structures-Discriminant Analysis (OPLS-DA) [29] models between WT and mutants to hierarchical cluster analysis and heat-map visualization [28]. Our results show that metabolite profiles of Mediator subunit mutants, which in biochemical and structural analyses in other organisms have been reported to reside in the same Mediator module, clustered together (Fig 1C). For example, *med17*, *med18*, and *med22a* formed a specific cluster and the corresponding proteins have all been shown to be part of the head module in yeast and mammalian cells [30].

Comparative genomic analyses in plant, yeast and metazoan cells have revealed homologies between the yeast (y) MED3 and Arabidopsis (a)/metazoan (m) MED27, the yMED2, mMED29, and aMED32, and the yMED5, mMed24, and aMED33A subunits [10]. The yMed2, yMed3, and yMed5 proteins have all been identified as subunits of the tail module [4, 31]. Accordingly, we found that the Arabidopsis *med27*, *med32*, and *med33a* mutants showed metabolite patterns that are similar to each other. Finally, we found that the *dreb2a* mutant, which we have reported to show phenotypes in response to drought, which are opposite to the *med25* mutant [17], also displays changes in metabolite levels which in most cases are opposite to the changes observed in *med25* (Fig 1C). Our results therefore imply that subunits which co-localize in the same Mediator module are involved in the regulation of genes involved in related metabolic pathways and that previously observed differences in phenotypes are reflected by differences in their metabolite patterns. Metabolic profiling of Mediator mutants therefore provide a complement to other methods for studying how Mediator subunits are localized relative to each other.

Based on the results described above, we examined the clustering analysis in order to suggest location of Mediator subunits that are unique to plants and/or higher eukaryotes, and have not been assigned to a specific Mediator module. We found that *med25* clusters with the head module mutants *i*.*e*. *med17*, *med18*, *med19a*, and *med22a* (Fig 1C). This is in line with our previous results, which show that *med25* displays phenotypes similar to the *med8* and *med18* head module mutants [16, 17]. To validate our metabolomic data, we performed pull-down experiments using Arabidopsis MED25 co-expressed with MED18 and MED20, which form a dimer in head [30]. In agreement with our metabolic profiling, we found that MED25 interacts with the MED18/MED20 dimer (Fig 1D), thus supporting the localization of MED25 to the head module. Finally, we found that *med28* and *med34* clustered with mutants of the tail module, that *med19a* clustered with mutants of the head module and finally that *med23* formed a separate cluster different from those of the head and tail subunits. These results indicate that different metabolic pathways are independently processed by each Mediator module and that predicted Mediator subunit interactions based on functional metabolomic analysis corroborate previous reports based on yeast 2-hybrid experiments, co-immunoprecipitation and phenotypic characterization of Mediator mutants [32].

### *med18* and *med25*, display changes in oxylipin-containing galactolipid levels

**Fig 2:**
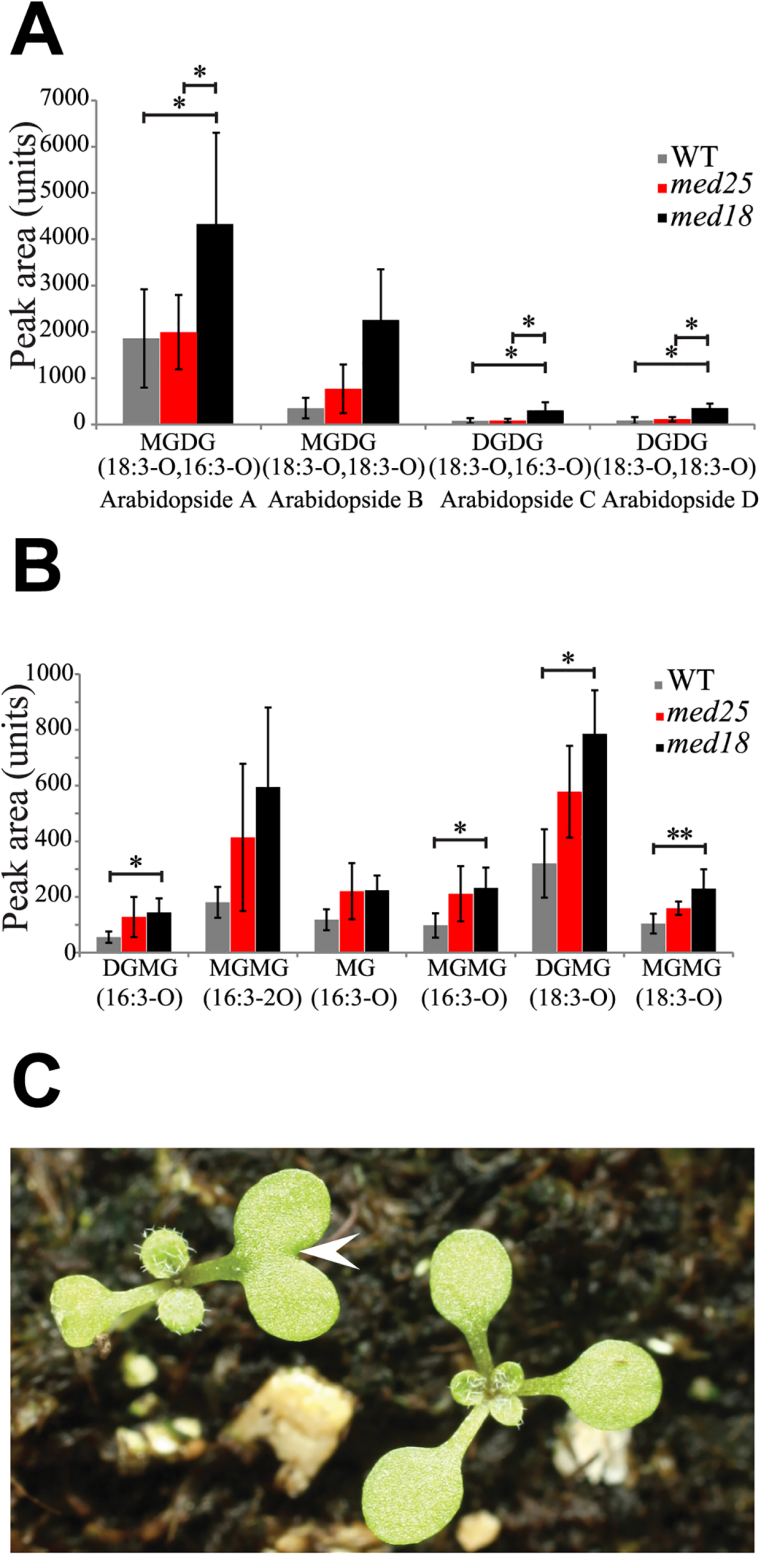
Oxylipin-containing galactolipids metabolite levels in *med18*, *med25*, and WT and a specific developmental phenotype for med18. (A) Levels of specific Arabidopsides in, *med18*, *med25*, and WT. (B) Levels of oxylipin containing lyso-galactolipids in *med18*, *med25*, and WT. (C) *med18* shows additional or fused cotyledons at the germination stage. ~20% of the seedlings show additional and/or fused cotyledons (arrowhead). Standard deviations were calculated from six biological repeats. Significance analyses of differences between WT and mutants was performed using an analysis of variance (ANOVA) with Tukey's HSD applied as a posthoc test (*, P ≤ 0.05; **, P ≤ 0.01).

Our metabolome and immunoprecipitation analyses suggested that MED18 and MED25 are located in the head module and therefore probably function in similar regulatory pathways. Indeed, both subunits are involved in pathogen defense as well as flowering-time regulation [19, 20, 33] (S1 Fig). To further explore the possibility of co-location-mediated functional similarity, we compared the *med18* and *med25* metabolite profiles in more detail. We identified *med18* and *med25* to have altered levels of oxylipin-containing galactolipids as compared to the WT. Galactolipids are the most abundant lipid in chloroplast membranes. A specific sub-class of galactolipids is called Arabidopsides, which are monogalatosyldiacylglyceride (MGDG), or digalactosyldiacylglycerol (DGDG) containing 12-oxo-phytodienoic acid (OPDA) or dinor (dn)-OPDA. Arabidopsides correspond to an accessible pool of oxylipins that provide precursors for JA synthesis during stress conditions. Depending on the distribution of OPDA and/or dn-OPDA at the sn1- and sn2-positions on the galactolipids, we could identify specific Arabidopside types [12]. The most representative Arabidopsides were A, B, C, and D (Fig 2A and Table 1). The *med18* mutant showed a significant increase of the Arabidopside A, C, and D levels, both compared to WT and *med25*. Our metabolomic analyses also revealed significantly higher levels of oxylipin-containing lysogalactolipids in *med18* and *med25* compared to WT (Fig 2B and Table 2).

**Table 1:** Levels of specific Arabidopsides in *med18*, *med25*, and *WT*.

	*med18*		*med25*		*WT*	
	Mean	Stdv	Mean	Stdv	Mean	Stdv
MGDG (18:3-O, 16:3-O) Arabidopside A	4,349	1,955	1,995	801,6	1,857	1,061
MGDG (18:3-O, 18:3-O) Arabidopside B	2,258	1,093	770.0	525.3	352.4	222.5
DGDG (18:3-O, 16:3-O) Arabidopside C	304.7	176.6	82.86	40.39	77.13	55.72
DGDG (18:3-O, 18:3-O) Arabidopside D	355.1	97.08	112.4	47.84	85.72	75.19

**Table 2:** Levels of specific oxylipin containing lyso-galactolipids in *med18*, *med25*, and WT.

	*med18*		*med25*		*WT*	
	Mean	Stdv	Mean	Stdv	Mean	Stdv
DGMG (16:3-O)	143.7	51.43	128.0	71.97	55.61	20.35
MGMG (16:3-2O)	594.5	285.6	413.8	264.5	180.6	55.54
MG (16:3-O)	223.6	52.91	220.6	100.8	118.3	37.56
MGMG(16:3-O)	232.9	72.05	211.4	98.91	97.68	43.64
DGMG (18:3-O)	785.8	155.9	577.5	164.7	320.4	122.7
MGMG (18:3-O)	230.1	68.89	159.5	23.86	104.3	35.77

To determine if the changes in metabolite levels in *med18* and *med25* are caused by differential expression of genes involved in their synthesis, we performed Affymetrix microarray analyses on leaf tissue collected at the same developmental stage (bolting time) as for the metabolomics experiments. Using a criterion of a 1.42-fold (log_2_ fold change >0.5 or <-0.5) difference, in combination with p-value of ≤ 0.05 between a mutant and WT to define significant changes in gene expression, we found that 652 and 384 genes were downregulated while 634 and 172 genes were upregulated in *med18* and *med25*, respectively (S7 and S8 Tables). Of these genes, 125 were downregulated and 39 were upregulated in both mutants, while 19 genes were differently regulated (S9 Table). Gene ontology analysis showed that genes whose expression is affected in both *med18* and *med25* encode proteins and enzymes involved in similar, but not identical pathways (S3 Fig).

We next analyzed the effects on expression of specific genes. Patatin-related Phospholipase A1 (*pPLA1*) uses Arabidopsides as substrates to release OPDA and dn-OPDA, and it was shown to promote resistance to the necrotrophic fungus *Botrytis cinerea* [34]. Another pPLA, pPLAIIα, which hydrolyses unoxidized and oxidized glycolipids, inhibits oxylipin production suggesting a role in removal of oxidized fatty acids from membranes. Suppression of pPLAIIα has been shown to confer resistance to fungal and bacterial infections [35]. We found that the expression of p*PLA1* was slightly increased in *med18* but not in *med25* (S7 and S8 Tables). In contrast, p*PLAII*α expression was reduced in both *med18* and *med25*, which could explain the higher levels of Arabidopsides in these mutants. Overexpression of TCP (TEOSINTE BRANCHED1, CYCLOIDEA, and PCF) TFs leads to increased JA biosynthesis, via regulation of oxylipin biosynthesis [36]. In our microarray experiments, we found that expression of *TCP3* was 1.4-fold higher in *med18* than in WT. We found that about 20% of the *med18* seedlings exhibited additional or fused cotyledons (Fig 2C), similar to *med20a*, *dcaf1*, and *cullin3* [37, 38]. Our microarray data therefore support the documented changes in oxylipin levels and the phenotypic effects recorded for *med18*.

### *med18* displays changed levels of phenolic metabolites

**Fig 3:**
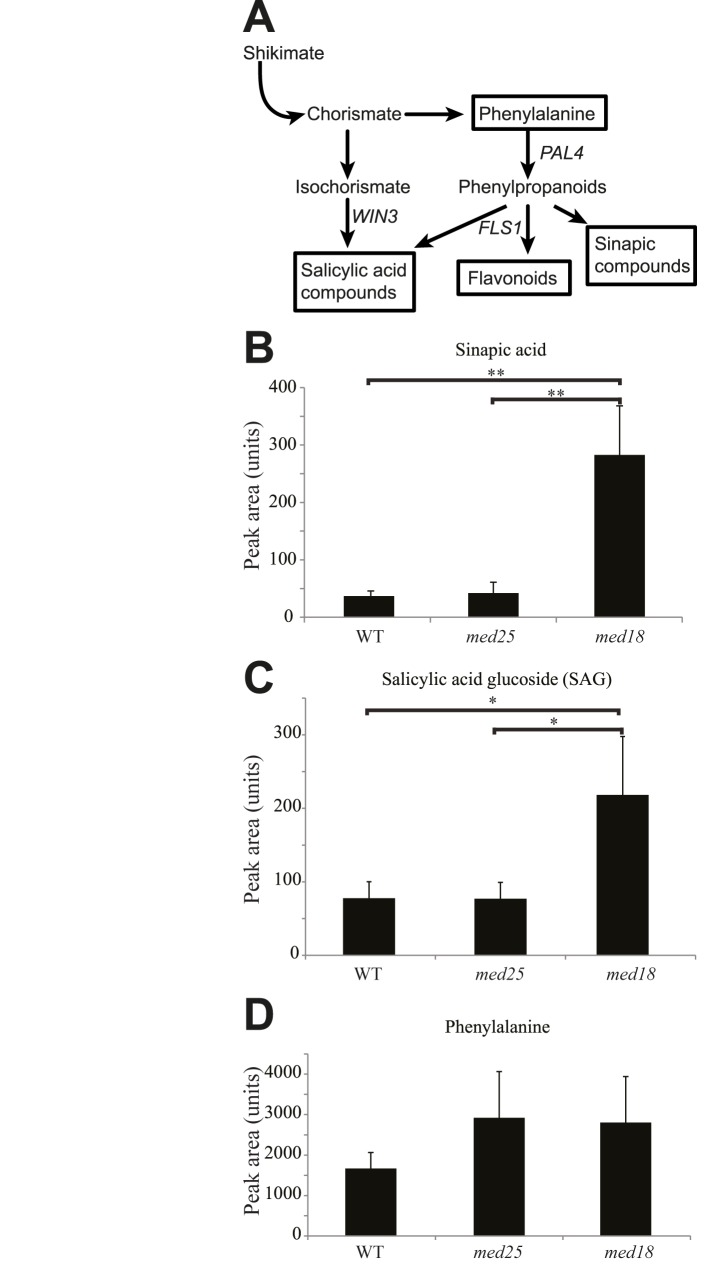
Phenolic metabolite levels in *med18*, *med25*, and WT. (A) Overview of the phenylpropanoid synthesis pathway. (B) Levels of sinapic acid. (C) Levels of salicylic acid glucoside (SAG). (D) Levels of phenylalanine. Standard deviations were calculated from six biological repeats. Significance analysis of differences between WT and mutant was performed using an analysis of variance (ANOVA) with Tukey's HSD applied as a posthoc test (*, P ≤ 0.05; **, P ≤ 0.01).

In further analysis of our metabolite data, we observed a 7-fold increase in sinapic acid levels and a 3-fold increase in salicylic acid glucoside (SAG) in *med18* compared to WT (Fig 3A–3C; S3–S6 Tables). Accordingly, several genes (At4g36220, At1g15950, At3g21560, At1g80820, At2g21890) encoding enzymes in the sinapoyl ester pathway were up-regulated in *med18* (S7 Table). SA compounds can be produced both by the isochorismate and phenylpropanoid pathways and phenylalanine is a common intermediate for SA, flavonoid, and sinapoyl biosynthesis (Fig 3A). We detected a tendency for increased levels of phenylalanine in *med18* and *med25* compared to WT, but it was not statistically significant (Fig 3D; S3–S6 Tables). Overall, our metabolomic experiments suggest an increase of metabolic flow towards SA and sinapoyl metabolites in *med18* (Fig 3A–3C). The *med18* mutant also showed a 1.5-fold induction of *PHENYLALANINE AMMONIA-LYASE 4* (*PAL4*) (S7 Table), which encodes one of four PALs in Arabidopsis and catalyzes the first step in the phenylpropanoid pathway. PAL activity is important for both SA and sinapate biosynthesis and regulates the interaction between primary and secondary metabolism. We also detected changes in expression levels of some genes acting in the isochorismate pathway *i*.*e*. a 2.0-fold increase expression of *WIN3* in *med18*, which confers resistance to the biotrophic pathogen *Pseudomonas syringae* (Fig 3A and S7 Table). The increased expression of *PAL4* and *WIN3* may explain the increased levels of SA compounds and sinapoyl metabolites in *med18*. Interestingly, a *med5a/med5b* double mutant was recently shown to have increased levels of sinapoyl metabolites and displayed homeostatic repression of phenylpropanoid biosynthesis in Arabidopsis [39].

### *med18* displays changes in glucosinolates and tryptophan-derived metabolite levels

**Fig 4:**
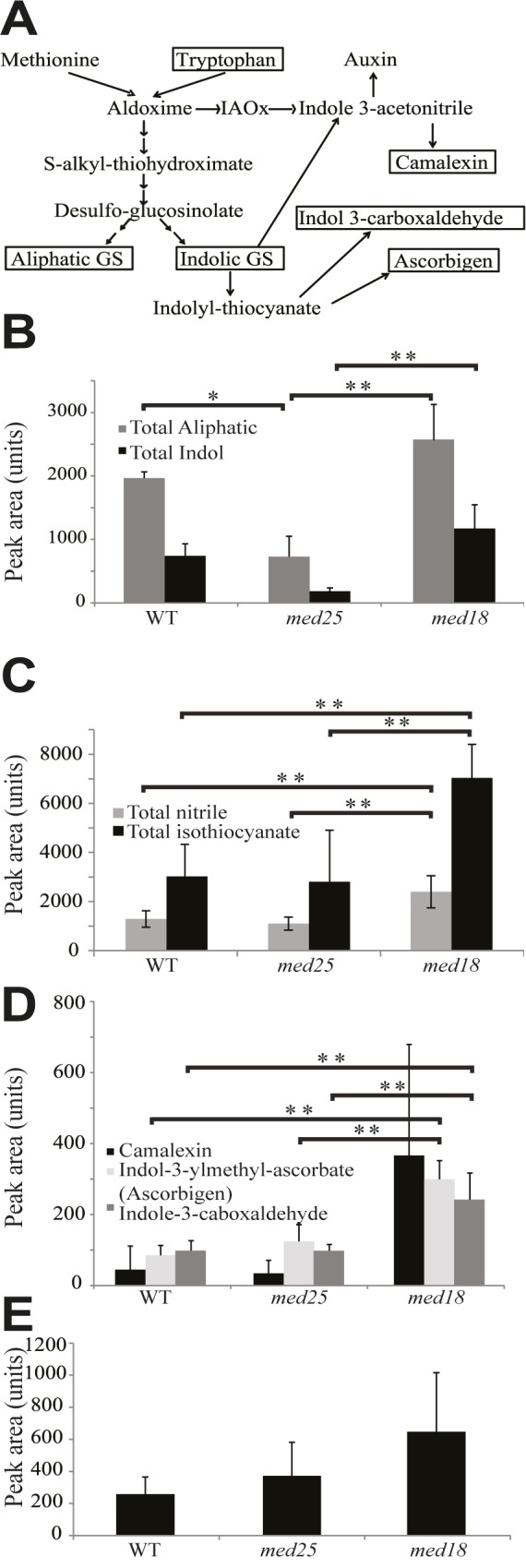
Glucosinolate and tryptophan-derived metabolite levels in *med18*, *med25*, and WT. (A) Overview of the glucosinolate metabolism and phytoalexin synthesis pathways. (B) Total levels of aliphatic (grey bars) and total indole glucosinolates (black bars). (C) Levels of aliphatic glucosinolate breakdown metabolites. Total nitrile (grey bars), total isothiocyanate (black bars). (D) Levels of indole glucosinolates breakdown metabolites. (E) Levels of tryptophan. Standard deviations were calculated from six biological repeats. Significance analysis of differences between WT and mutant was performed using an analysis of variance (ANOVA) with Tukey's HSD applied as a posthoc test (*, P ≤ 0.05; **, P ≤ 0.01).

Glucosinolates can be derived from methionine or tryptophan (Fig 4A) and are consequently classified as aliphatic or indolic glucosinolates, respectively, where aliphatic glucosinolates are the most abundant in Arabidopsis [40]. We found that the levels of both total aliphatic and total indolic glucosinolates were increased in *med18*, but decreased in *med25* (Fig 4B and S10 Table). Intact glucosinolates are biologically inactive, but after hydrolyzation by myrosinases their breakdown products have roles in insect and pathogen defense [41]. Glucosinolates and myrosinases normally localize in different cellular compartments but co-localize after mechanical disruption. Even under non-stressed conditions, we found that *med18* contained >2-fold increased levels of both aliphatic and indolic glucosinolate degradation products (Fig 4C and 4D and S11 Table). Aliphatic and indolic isothiocyanates are produced by spontaneous reactions and they are reported to be crucial to combat pathogenic infections in Arabidopsis [42]. Their increased levels in *med18* might thus contribute to its increased pathogen resistance [20]. We also found that *med18* displayed an increase in isothiocyanate and nitrile breakdown products (S11 Table). This correlated with our observation that genes involved in the methionine glucosinolates pathway are up-regulated in *med18* (*i*.*e*. *CYP79F1*, 1.6-fold and Glucosinolate S-oxygenase, 2-fold; S7 Table).

Indole glucosinolates are produced from the conversion of tryptophan to indole-3-acetaldoxime (IAOx), a key branching point between several secondary metabolites [41]. We detected increased tryptophan levels in *med18* (Fig 4E and S6 Table), which may contribute to the 2-fold higher levels of indole glucosinolates in this mutant (Fig 4B and S6 Table). Camalexin produced from IAOx is the major indole phytoalexin in Arabidopsis and is involved in defense against a wide range of pathogens [43]. Our results show that the levels of camalexin were increased in *med18* (Fig 4D). Camalexin levels are normally very low in Arabidopsis, but increase ~100-fold in response to microorganisms and abiotic stress conditions. In addition, other indole phytoalexin products like ascorbigen (indole-3-carbinol ascorbate) and indole 3-carboxaldehyde were increased in *med18* (Fig 4D). Based on the increased levels of aliphatic and indolic glucosinolates, and their breakdown products in uninfected cells, we conclude that *med18* in several ways is metabolically prepared to combat a pathogenic attack already before it has occurred.

## Discussion

**Fig 5:**
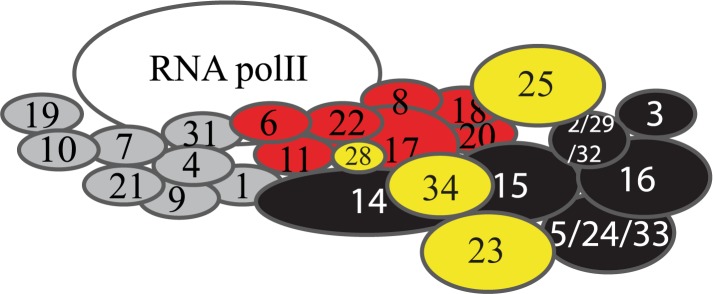
Proposed model for localization of the Arabidopsis Mediator subunits Med23, Med25, Med28 and Med34 based on the hierarchical cluster analysis and heat-map visualization presented in Fig 1C. Labeling of subunits is as described in Fig 1C. Subunits in yellow represent subunits whose localization is suggested based on the results presented here.

Despite an increasing number of reports on Mediator in plants since its original identification in 2007 [9] we still have a relatively limited knowledge about its structure and function. Here, we integrate metabolome, transcriptome, and functional analyses to identify new roles for subunits of the plant Mediator complex. By applying hierarchical cluster analysis of the metabolite levels in Mediator subunit mutants, we found that mutants representing subunits of the same module cluster together, indicating that related metabolic pathways are processed by the same Mediator module. Our metabolomic analyzes showed that he *med27*, *med32* and *med33a* mutants grouped together with the *med22b*, *med28* and *med34* mutants. The Arabidopsis MED27, MED32 and MED33a are reported as homologous to the yeast MED3, MED2 and MED5 subunits respectively, which have all been localized to the tail domain by a combination of biochemical, genetic and structural methods [4–7]. Similarly, we found that the *med17*, *med18*, and *med22a* mutants grouped with the *med19a* and *med25* mutants. MED17, MED18 and MED22 are defined as head module subunits in yeast. Finally, the *med23* mutant showed a metabolite profile that differed from mutants in genes encoding head or tail subunits. Accordingly, our metabolite profiling suggest that MED28 and MED34 are located in the tail module, MED19a and MED25 in the head module while the unassigned Arabidopsis MED23 might be part of the middle module. As an independent method, we confirmed the localization of MED25 to the head module by pull-down experiments using recombinant MED25 and head module subunits MED18 and MED20. In contrast to our findings here, MED25 was recently reported to interact with Med16 in Arabidopsis [44] and with subunits of the tail module in human cells based on results from a combination of cryo-EM mapping and immunoprecipitation experiments using epitope-tagged Mediator subunits [6]. However, this study also provided a revised model for how different Mediator modules are located in relation to each other. Previous reports suggested that Mediator had an elongated shape where head was located closest to Pol II and tail was located farthest away with Middle as a bridging module. In the new model, head and tail are adjacent and make direct contact to each other. This is in agreement with our functional metabolomic results, which show that the metabolite levels in head and tail subunit mutants are more similar to each other compared to those observed in middle subunit mutants (Fig 1C). Even if no experiments aimed at revealing or disproving interactions between MED25 and subunits of the head or middle modules were presented, the updated model where tail and head are more adjacent than previously anticipated makes MED25 interactions to both modules possible. A model for this interpretation of our results are presented in Fig 5.

Despite overall similarity in their metabolomic signature, our results show that *med18* and *med25* display unique levels of oxylipin-containing galactolipids, phenolic metabolites, glucosinolates, and camalexin. These metabolites have all been implicated in responses to abiotic stress, pathogen infections, and in development [45, 46]. The most understood functional aspects of the oxylipin pathways in plants originate from studies of JA, its precursor OPDA and their derivatives, collectively called jasmonates. They have key signaling roles as hormones in transduction pathways that regulate expression of genes which control growth, photosynthesis and flowering [47]. The atypical OPDA-galactolipid levels and the increased levels of certain Arabidopsides might explain some of the phenotypes that we report here.

In particular, we found that *med18* contains increased levels of SA compounds, sinapic acids, and glucosinolates; metabolites known to be involved in responses to pathogens. It has been reported that exogenous treatment with SA has a beneficial effect on *F*. *oxysporum* resistance [48]. However, induced levels of glucosinolates have also been implicated in resistance to different types of pathogens, which indicate a correlation between their levels and the degree of colonization of *F*. *oxysporum* in *med18* roots [41, 42]. Also the Arabidopsis *gsm1-1* mutant, which has reduced aliphatic glucosinolates, shows enhanced susceptibility to Fusarium [49]. The *gsm1-1* mutant contained reduced levels of 4-methylsulphinylbutyl isothiocyanate (sulphoraphane) and sulphoraphane demonstrated toxicity to *F*. *oxysporum in vitro*. In line with these observations, we found an increase in sulphoraphane and other aliphatic glucosinolate breakdown products in *med18* relative to WT (S11 Table) suggesting that sulphoraphane and potentially other compounds may play a role in the increased resistance to *F*. *oxysporum*.

We also noticed that the camalexin levels were higher in un-inoculated *med18* plants compared to WT, which is interesting since camalexin is the major phytoalexin in Arabidopsis and is generally present at low levels in unchallenged plants [43]. Fusarium has been shown to be sensitive to camalexin in *in vitro* plate assays [50]. Camalexin levels have also been found to increase in response to the root pathogen *Verticillium longisporum* and were found to inhibit *V*. *longisporum in vitro* [51]. Our results suggest that increased basal levels of camalexin and glucosinolate breakdown products may act as a chemical barrier to prevent *F*. *oxysporum* from colonizing *med18* plants. In support of our results presented here, an accompanying paper describe that both *med18* and *med20* mutants show a strong level of resistance to Fusarium infection in Arabidopsis [20].

MYC2 and the related bHLH TFs, MYC3, and MYC4, are known to regulate the TRP pathway and the production of glucosinolates [52] and a recent study revealed that the *myc2*/*myc3*/*myc4* triple mutant was almost completely devoid in glucosinolates [53]. We observed no increased expression of *MYC3*, *MYC4*, or other TFs that might cause increased glucosinolate levels in *med18*. Therefore further investigation of the TFs that are altered in *med18* is required. A recent study reported that *med18* is more susceptible to the necrotrophic pathogen *Botrytis cinerea* and causes the TF, YIN YANG1, to suppress expression of glutaredoxins *GRX480*, *GRXS13*, and thioredoxin *TRX-h5* [54]. We have yet to explore the role of these genes in Fusarium resistance; however the susceptibility of *med18* to leaf necrotrophs such as *B*. *cinerea* is similar to other *F*. *oxysporum* resistant mutants, *coi1*, *med25*, and *med8* which are susceptible to *Alternaria brassicicola* and *B*. *cinerea* suggesting an additional link between *F*. *oxysporum* resistance and susceptibility to necrotrophs.

Some of the changes in metabolite levels and expression of specific genes that we detect in *med18* can be linked to specific developmental phenotypes. Indeed, we found that some *med18* seedlings displayed additional and/or fused cotyledons suggesting that MED18 is involved in signal pathways that regulate embryogenesis. *CUC1* and *CUC2* encode key TFs involved in organ boundary specification, formation of fused cotyledons, pleiotropic phyllotaxy phenotypes, and increased oxylipin levels [55]. These findings might contribute to some of the phenotypes that we observe for *med18* here.

Our results shed light on functions of Mediator in higher eukaryotes and indicate that different Mediator modules are involved in specific metabolic pathways that control developmental programs and responses to pathogen infections. Our findings also show that functional metabolomics can be used as a complement to other methods in order to reveal how subunits of multiprotein complexes interact and provide a base for further studies on Mediator subunit function in the integration of signals from different environmental cues in order to elicit a functional cellular response.

## Supporting information

S1 Fig Analysis of different developmental phases in Mediator mutants and *dreb2a*. Growth stage progression was determined in LD conditions. Arrows define the time after sowing at which WT plants reached the indicated growth stages. Boxes represent the time elapsed between the occurrences of each successive growth stage. Days are given relative to date of sowing, including a 1-day stratification at 4°C to synchronize seed germination. Data are given as averages ± SD for 4 individual experiments.
(EPS)

S2 Fig Putative metabolites used to cluster the mediator subunits in three groups: Head, middle and tail. The peak number (putative metabolite) corresponds to peak information in S3 Table.
(PDF)

S3 Fig Gene Ontology (GO) analysis for biological function. (A) Bars represent the percentage of the 652 and 384 down-regulated genes in *med18* and *med25* that fall into the indicated GO categories. (B) Bars represent the percentage of the 634 and 172 up-regulated genes in *med18* and *med25* that fall into the indicated GO categories.
(EPS)

S1 Table Position, status and mRNA expression levels in the T-DNA insertion lines. (DOCX)

S2 Table Oligonucleotides used for RT-PCR. (DOCX)

S3 Table Relative abundance of metabolites detected by LC-TOF MS (+) in WT, *dreb2a* and 12 arabidopsis mediator subunit mutant lines. (XLSX)

S4 Table Metabolite grouping extracted from the global dataset as visualized in the heat map in Fig 1C. (XLSX)

S5 Table Metabolite annotation from the metabolomic analysis performed in WT, *dreb2a* and 12 arabidopsis mediator subunits mutant lines by LC TOF MS (+). (XLSX)

S6 Table Glucosinolates and breakdown products identified in the experiments by LC-MS in negative or positive mode. (XLSX)

S7 Table Affymetrix microarray analysis of *med18*. (XLSX)

S8 Table Affymetrix microarray analysis of *med25*. (XLSX)

S9 Table Genes that are differently regulated in both *med18* and *med25* relative to WT. (XLSX)

S10 Table Levels of specific glucosinolates in *med18*, *med25*, and WT. (DOCX)

S11 Table Levels of specific glucosinolate breakdown products in *med18*, *med25* and WT. (DOCX)
