# Supplementary figures and images for: Functional metabolomics as a tool to analyze Mediator function and structure in plants
Source: PLoS One. 2017 Jun 22;12(6):e0179640. doi: 10.1371/journal.pone.0179640 (PMC5480960; doi:10.1371/journal.pone.0179640)

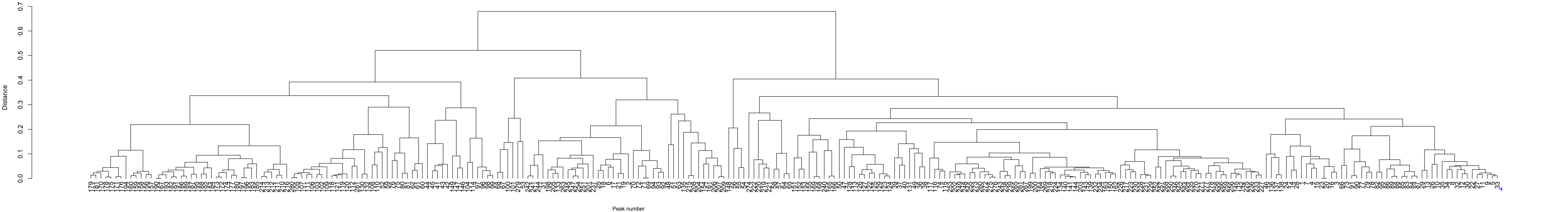

Supplement: S2 Fig — The peak number (putative metabolite) corresponds to peak information in S3 Table. (PDF) [file pone.0179640.s002.pdf]
