# Supplementary material for: Functional metabolomics as a tool to analyze Mediator function and structure in plants
Source: PLoS One. 2017 Jun 22;12(6):e0179640. doi: 10.1371/journal.pone.0179640 (PMC5480960; doi:10.1371/journal.pone.0179640)
Supplement: S1 Table — (DOCX) [file pone.0179640.s004.docx]

| **Protein** | **AGI** | **NASC** | **T-DNA** | **Status** | **Position** | **mRNA expression levels relative to WT** |
| --- | --- | --- | --- | --- | --- | --- |
| N/A | N/A | Col-0 | N/A | N/A | N/A | N/A |
| Med17 | AT5G20170 | N602813 | SALK_102813 | Heteroz | Exon | 50% |
| Med18 | AT2G22370 | N527178 | SALK_027178 | Homoz | Intron | <10% |
| Med19a | AT5G12230 | N658182 | SALK_034955C | Homoz | Intron | <10% |
| Med22a | AT1G16430 | N662709 | SALK_063109C | Homoz | 5´ | 70% |
| Med22b | AT1G07950 | N656094 | SALK_001024C | Homoz | Intron | <10% |
| Med23 | AT1G23230 | N628011 | SALK_128011 | Homoz | Intron | <10% |
| Med25 | AT1G25540 | N629555 | SALK_129555 | Homoz | Intron | <10% |
| Med27 | AT3G09180 | N512449 | SALK_012449 | Homoz | 5´ | 60% |
| Med28 | AT3G52860 | N537570 | SALK_037570 | Homoz | 5´ | 75% |
| Med32 | AT1G11760 | N665553 | SALK_023845C | Homoz | Intron | <10% |
| Med33a | AT3G23590 | N589976 | SALK_089976 | Homoz | Exon | <10% |
| Med34 | AT1G31360 | N587178 | SALK_087178 | Homoz | Exon | <10% |
| Dreb2a | AT5G05410 | N873547 | SAIL_365_F10 | Homoz | Exon | <20% |

**Supplementary Table S1. Position, status and mRNA expression levels in the T-DNA insertion lines**
