# Supplementary material for: Functional metabolomics as a tool to analyze Mediator function and structure in plants
Source: PLoS One. 2017 Jun 22;12(6):e0179640. doi: 10.1371/journal.pone.0179640 (PMC5480960; doi:10.1371/journal.pone.0179640)
Supplement: S2 Table — (DOCX) [file pone.0179640.s005.docx]

**Target gene Sequence Primer used for Direction Annealing temperature (⁰C)**

Med18 CGAACCCACATGGACGGTTA RT-PCR forward 57

ACAACCCCCGTGGAGATAGA RT-PCR reverse 57

Med19a TGAGAGCATATCACAGAGCC RT-PCR forward 57

GAATGATCACCCGAATCATGG RT-PCR reverse 57

Med22b CACGCAGATCGTCAATGTTTC RT-PCR forward 57

GCCGTTTCCTCTGGTTTATG RT-PCR reverse 57

Med23 CCGGCCTGTGACATCTGTGGC RT-PCR forward 57

GGGCTGTTGTCTGTGCGGTC RT-PCR reverse 57

Med25 TCTTGCATTGGCTTTCTTCCA RT-PCR forward 57

TGGAACTGGTCCAACAGAAAC RT-PCR reverse 57

Med32 TGACACAAATCGGACTCTCC RT-PCR forward 55

CCACGTTGCAGCTCAAGAAC RT-PCR reverse 55

Med33a TTCTTGCAACAACAGGCGTC RT-PCR forward 55

TCCACGCCCCAAGTAAGT RT-PCR reverse 55

Med34 TCGTCGAAGCGCATTTTTCC RT-PCR forward 57

GAGCCAATCTGCTGGGTTGA RT-PCR reverse 57

Dreb2a CGAAAGCGATTTATCAACTCG RT-PCR forward 55

AAACACATCGTCGCCATTTAG RT-PCR reverse 55

Med17 GTGATGATTCTCCAACAGGTG RT-qPCR forward 61

TTTGCGTCTTCCGGGTTTACA RT-qPCR reverse 61

Med22a ACCAACAGCTGCTGCGGCA RT-qPCR forward 62

GCTGCTTGAACCAATCTTGATG RT-qPCR reverse 62

Med27 GCTCTCTACTCTCTTCTGCT RT-qPCR forward 57

ATTCTTGGTAAGCGCGGTGT RT-qPCR reverse 57

Med28 AATTGATGTGGAGCGGCACG RT-qPCR forward 59

ACTGCAATATCCTTCTTAAGGC RT-qPCR reverse 59

Actin2 AATTACCCGATGGGCA RT-PCR forward 52

TCATACTCGGCCTTGGA RT-PCR reverse 52

Actin2 GTCGTTGCACCACCTGAAAG RT-qPCR forward 57-62

CCTTGGAGATCCACATCTGCT RT-qPCR reverse 57-62

Eif4 TTGTCAACACTCGTCGCAAGGT RT-qPCR forward 57-62

AGCCAGACCTGAACTCTCTCATGA RT-qPCR reverse 57-62
