# Supplementary material for: Functional metabolomics as a tool to analyze Mediator function and structure in plants
Source: PLoS One. 2017 Jun 22;12(6):e0179640. doi: 10.1371/journal.pone.0179640 (PMC5480960; doi:10.1371/journal.pone.0179640)
Supplement: S10 Table — (DOCX) [file pone.0179640.s013.docx]

| **Glucosinolate** | ***med18*** | | ***med25*** | | **WT** | |
| --- | --- | --- | --- | --- | --- | --- |
|  | Mean | Stdv | Mean | Stdv | Mean | Stdv |
| 3-methylsulfinyl-propyl | 46.8 | 10.8 | 32.8 | 17.2 | 49.5 | 7.50 |
| 4-methylsulfinyl-butyl | 1730 | 400 | 638 | 234 | 1260 | 287 |
| 5-methylsulfinyl-pentyl | 59.5 | 10.5 | 26.4 | 6.20 | 43.0 | 2.00 |
| 7-Methylsulfinyl-heptyl | 53.2 | 12.3 | 25.2 | 7.30 | 104 | 25.5 |
| 8-methylsulfinyl-octyl | 672 | 165 | 101 | 61.3 | 512 | 26.5 |
| 1-methoxyindol-3-ylmethyl | 32.5 | 11.6 | 25.8 | 4.10 | 83.5 | 22.5 |
| 3-Indolylmethyl | 1070 | 404 | 106 | 55.7 | 294 | 61.0 |
| 4-hydroxy indole 3-yl-methyl | 69.0 | 28.4 | 51.4 | 8.70 | 363 | 107 |

**Supplementary Table S10: Levels of specific glucosinolates in *med18*, *med25*, and WT**
