# Supplementary material for: Functional metabolomics as a tool to analyze Mediator function and structure in plants
Source: PLoS One. 2017 Jun 22;12(6):e0179640. doi: 10.1371/journal.pone.0179640 (PMC5480960; doi:10.1371/journal.pone.0179640)
Supplement: S11 Table — (DOCX) [file pone.0179640.s014.docx]

| **Breakdown products** | ***med18*** | | ***med25*** | | **WT** | |
| --- | --- | --- | --- | --- | --- | --- |
|  | Mean | Stdv | Mean | Stdv | Mean | Stdv |
| 8-(Methylsulfinyl)octanenitrile | 335 | 73.2 | 335 | 46.2 | 296 | 38.6 |
| 9-(Methylsulfinyl)nonanenitrile | 1540 | 482 | 525 | 154 | 701 | 193 |
| 7-Methylsulphinylheptyl nitrile | 522 | 97.5 | 243 | 70.3 | 292 | 102 |
| 4-methylsulfinylbutylthiocyanate (Sulphoraphan) | 1690 | 407 | 1020 | 324 | 822 | 195 |
| 8-Methylsulfinyloctyl isothiocyanate | 5350 | 1680 | 1790 | 983 | 2200 | 1180 |

**Supplementary Table S11:** Levels of specific glucosinolate breakdown products in *med18*, *med25* and WT.
